# Electrochemical Hydrogen-Charging Effect on the Microstructural Evolution and Mechanical Behavior of Deformed Ti Alloy

**DOI:** 10.3390/ma18030518

**Published:** 2025-01-23

**Authors:** Hyojoo Lee, Sam Yaw Anaman, Jeong-Mook Choi, Leeju Park, Keunho Lee, Jae-Kook Kim, Jong-Sook Lee, Jae Yong Song, Joon-Sik Park, Hoon-Hwe Cho

**Affiliations:** 1 Department of Materials Science and Engineering, Hanbat National University, 125 Dongseodae-ro, Yuseong-Gu, Daejeon 34158, Republic of Korea; leehyojoo725@naver.com (H.L.);; 2 Technology Research Institute, Jinhap Co., Ltd., 40-57 Daehwa-dong, Daejeon 34302, Republic of Korea; 3 Agency for Defense Development, 160 Bugyuseong-daero 488 Beon-Gil, Yuseong-Gu, Daejeon 34186, Republic of Korea; 4 School of Materials Science and Engineering, Chonnam National University, Gwangju 61186, Republic of Korea; 5 Department of Semiconductor Engineering, Pohang University of Science and Technology, Pohang 37673, Republic of Korea

**Keywords:** Ti alloys, electrochemical hydrogen charging, hydrides, microstructure, mechanical properties, hydrogen diffusion

## Abstract

In this work, hydrogen is introduced into deformed Ti alloys via cathodic electrolysis under various charging conditions, and the microstructures and mechanical properties of the specimens are examined. The presence of hydrides is verified using X-ray diffraction, scanning electron microscopy, and electron backscatter diffraction analysis based on a rescanning approach using local pattern averaging. Hydrogen charging induces changes in the microstructure, making it possible to determine the hydrogen penetration depth. Vickers hardness tests are then performed to assess the extent of hydrogen penetration in the Ti alloys. Compression tests are conducted to further understand the impacts of hydrides on the mechanical reliability of hydrogen-charged Ti alloys. To reinforce the experimental findings, numerical simulations are conducted utilizing experimental data to comprehend hydrogen diffusion in the Ti alloys. Subsequently, the hydrogen diffusion mechanism in the hydrogen-charged specimens is verified through the obtained results.

## 1. Introduction

Ti alloys are extensively used across various industries, including the aerospace, marine, chemical, and biomedical fields, because of their exceptional strength-to-weight ratio, mechanical properties, and corrosion resistance [1,2,3]. The excellent corrosion resistance of a Ti alloy is attributed to the formation of a stable oxide layer, which is known as a passive film. This passive film effectively protects the Ti alloy from corrosion under diverse and harsh environmental conditions. However, Ti alloys are highly reactive with hydrogen [4,5]. Hydrogen reacts with many transition metals and alloys at high temperatures to form hydrides. Among electronegative elements, such as Sc, Yt, Ti, V, and Cr, Ti exhibits higher hydrogen reactivity than V and Cr, according to refs [6,7]. Under certain temperatures and pressures, Ti tends to absorb substantial amounts of hydrogen, thus forming hydrides that render them prone to hydrogen embrittlement (HE). HE can significantly reduce the impact strain energy of Ti alloys. Even trace amounts of hydrogen can induce HE and significantly challenge the practical applicability of Ti alloys [8,9]. Hence, it is crucial to comprehensively understand the formation of hydrides in Ti alloys, such as Ti-6Al-4V (Ti64) alloys, to effectively mitigate or prevent their occurrence.

The dual-phase alloy Ti64 is widely employed in industrial applications. This alloy comprises 6 wt.% aluminum, stabilizing the α phase, and 4 wt% vanadium, stabilizing the β phase [10,11]. Typically, the microstructure of Ti64 alloy can be altered through heat treatment, in which the cooling rate is critical in controlling the content of the β phase. At slow cooling rates in the α+β region or above the β transus temperature, the β phase is predominantly transformed into the equiaxed α phase. Increasing the cooling rate promotes the nucleation and growth of the α phase within the β crystal structure, resulting in a microstructure with dual lamellar morphology; with this morphology, the retained β phase exists as a Widmanstätten structure between α phase particles. The amounts and morphologies of the α and β phases and the densities of α/β interfaces significantly impact the mechanical and corrosion resistance properties of the alloy. Consequently, hydride formation and hydrogen diffusion in Ti64 alloy are contingent upon the phase features [12,13].

There are several forms of Ti-hydrides. At room temperature, quasi-stable hydrogen in γ-TiH_x_ (x < 1.5) consists of a face-centered tetragonal (FCT) crystal lattice, with c/a > 1. Conversely, δ-TiH_x_ (1.5 < x < 1.99) forms a face-centered cubic (FCC) crystal lattice. ε-TiH_x_ (x > 1.99) consists of an FCT with c/a < 1, and it is transformed without diffusion as the hydrogen content increases [14,15]. In environments containing hydrogen, substantial amounts of hydrogen can be absorbed and stored in the lattice of the Ti alloy, forming brittle hydrides (TiH_x_, where 0 < x ≤ 2) that could make the alloy prone to fracture. Even trace amounts of hydrogen could make Ti alloys less ductile or brittle [16]. Specifically, the lattice of Ti-α undergoes considerable volume expansion due to hydride formation, causing significant stress concentration in the Ti matrix and crack initiation. Almost all Ti alloys exhibit varying degrees of hydrogen susceptibility, posing significant challenges to their practical applications. Therefore, research on methods to suppress hydrogen-induced cracking (HIC) in Ti alloys is significantly required to bridge the research gap [17,18].

Therefore, research on methods for suppressing HIC tendencies in Ti alloys is highly important. Certain studies, such as that by Pan et al. [19], have demonstrated that alloy processing can effectively improve the mechanical properties of Ti alloys by adjusting their microstructures and crystal structures [20]. At room temperature, the α phase has very low hydrogen solubility, leading to hydrogen ingress, hydride formation, and HIC, even with very limited hydrogen uptake [21,22]. However, despite extensive efforts and significant advancements regarding the prevention of HIC and the regulation of hydrogen ingress and internal interactions, hydrogen-induced failures are of significant concern in many industrial applications [23,24]. Moreover, components comprising Ti alloys in marine environments are vulnerable to attack by hydrogen atoms, thus exposing them to HIC [25].

In hydrogen research, gas charging is commonly used to introduce hydrogen into the microstructures of materials. Among the mechanisms frequently employed for hydrogen charging, the electrochemical hydrogen charging or cathodic charging method is highly detrimental [26,27]. Molecular hydrogen, which is too large to enter the solid metal through the surface, must dissociate into atomic hydrogen during gas charging. Therefore, throughout electrochemical charging, hydrogen exists as protons in the electrolyte, and it can be adsorbed onto the metal surface as hydrogen atoms [27,28] These adsorbed hydrogen atoms can interact with the metallic matrix, leading to microstructural changes. However, according to previous studies [18,29], such changes are generally not significantly observed at room temperature, which can be attributed to the limited diffusion under these conditions.

Therefore, it is imperative to possess a thorough comprehension, estimation, and anticipation of hydrogen diffusion behavior during infiltration. Consequently, the utilization of numerical models, grounded in the diffusion characteristics of the Ti alloy, becomes crucial for predicting the depth of hydrogen diffusion, playing a pivotal role in quantifying the patterns over specific charging times. Research into the numerical modeling of hydrogen diffusion has emphasized the significance of juxtaposing the depth of hydrogen diffusion obtained through experimental approaches at specific times with that derived according to charging time. The overarching concept revolves around the validation of numerical models through experimental methods and leveraging these models to forecast hydrogen diffusion dynamics for adjusted parameters. These numerical models are applied using COMSOL Multiphysics to track the hydrogen diffusion and depth over time.

Hence, this study employs electrochemical hydrogen charging to explore the HIC behaviors exhibited by Ti alloys. The microstructures of the Ti alloy specimens subjected to hydrogen charging are examined, revealing notable structural changes even under room temperature conditions. The influence of hydrogen on their mechanical properties is also investigated. Subsequently, the experimental data are extracted and utilized in a numerical model to gain a deeper comprehension of hydrogen diffusion within the Ti alloys. This approach not only provides insights into the diffusion behavior but also highlights the microstructural transformations induced by hydrogen at ambient conditions. The verified hydrogen diffusion mechanism in the hydrogen-charged specimens is then established based on the obtained results.

## 2. Materials and Experimental Methods

### 2.1. Manufacturing Process and Specimen Preparation

**Figure 1:**
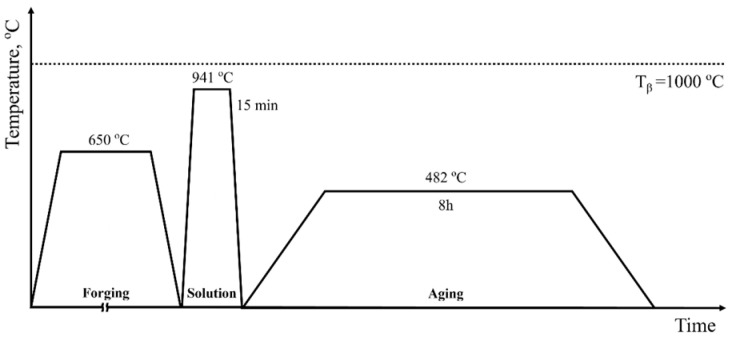
Schematic diagram of the thermomechanical processing for fabricating the fastener. Dotted line shows β-transus temperature (1000 °C).

**Figure 2:**
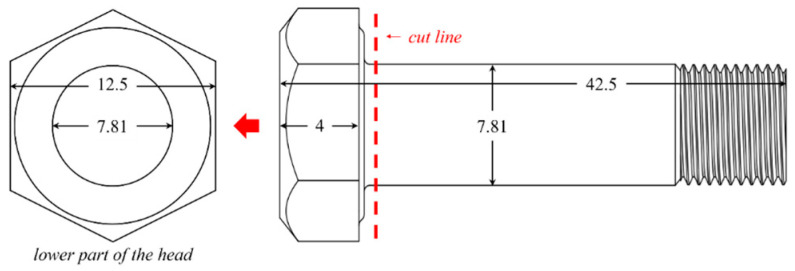
Schematic diagram and dimensions (units: mm) of the fastening bolt. The head of the fastening bolt is sectioned and used as the specimen for the electrochemical hydrogen charging.

Table 1 shows the composition of the Ti64 alloy used in this study. Initially, the Ti64 alloy was processed into a wire with a diameter of 7.81 mm through wire drawing and annealing. Subsequently, the wire was manufactured into various fastening components through hot-forming at 650 °C; the processing heat treatment conditions are shown in Figure 1. Due to excessive deformation during the forming of fastening components, in this study, the specimens will be referred to as ‘severely deformed Ti alloy’. Test specimens used in this study were prepared by wheel-cutting the head portion of the fastener—a hexagonal bolt—resulting in a specimen length of 12.5 mm (Figure 2), which underwent further sample preparations for the subsequent tests.

### 2.2. Electrochemical Hydrogen Charging

**Figure 3:**
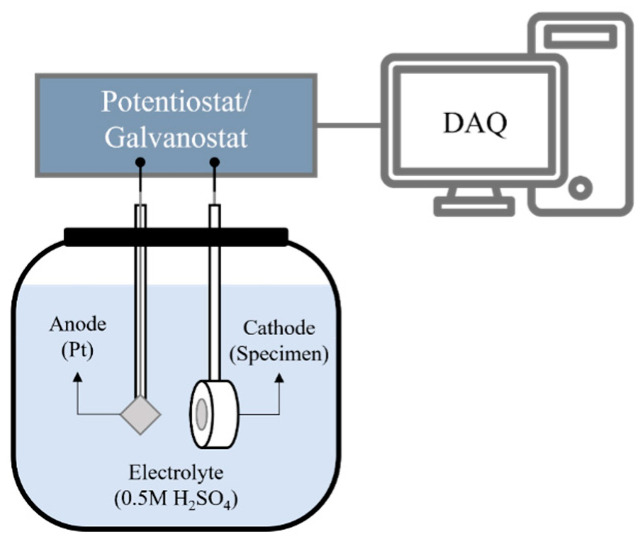
Schematic diagram of the experimental setup of electrochemical hydrogen charging. The setup is composed of Pt and specimen as anode and cathode, respectively. (DAQ: data acquisition).

Hydrogen charging was performed using the cathodic electrolytic method, where hydrogen generated at the cathode was forcibly injected into the specimen. As shown schematically in Figure 3, the test was conducted in a flat cell, with the specimen serving as the cathode and the Pt mesh serving as the anode. The hydrogen charging setup employed an electrochemical measurement device (potentiostat/galvanostat, ZIVE SP2, Seoul, South Korea). The electrolyte used in each test was 1000 mL of 0.5 M sulfuric acid solution (H_2_SO_4_) solution. The hydrogen charging conditions in the experiment were set with a constant current density of 100 mA/cm^2^ at room temperature and the potential is kept constant, preventing any galvanic effects. Additionally, the charging time served as the experimental parameter, varying from 0 to 4, 12, 24, and 48 h.

The cathodic electrolytic method is a type of water-splitting system, in which the oxygen evolution reaction (OER) occurs at the anode and the hydrogen evolution reaction (HER) occurs at the cathode. The overall water-splitting reaction could be represented as follows, depending on the acidic conditions of the electrolyte [30]:

Acidic conditions:

OER: 2H_2_O → O_2_ + 4H^+^ + 4e^−^ (1)

HER: 2H^+^ + 2e^−^ → H_2_ (2)

The hydrogen electrode reactions included the hydrogen oxidation reaction (HOR) at the anode and the HER at the cathode [31]. The HER occurred through the following steps under acidic conditions. 

Acidic conditions:

e^−^ + H^+^ → H_ad_ (Volmer reaction) (3)

H^+^ + H_ad_ + e^−^ → H_2_ (Heyrovsky reaction) (4)

**Figure 4:**
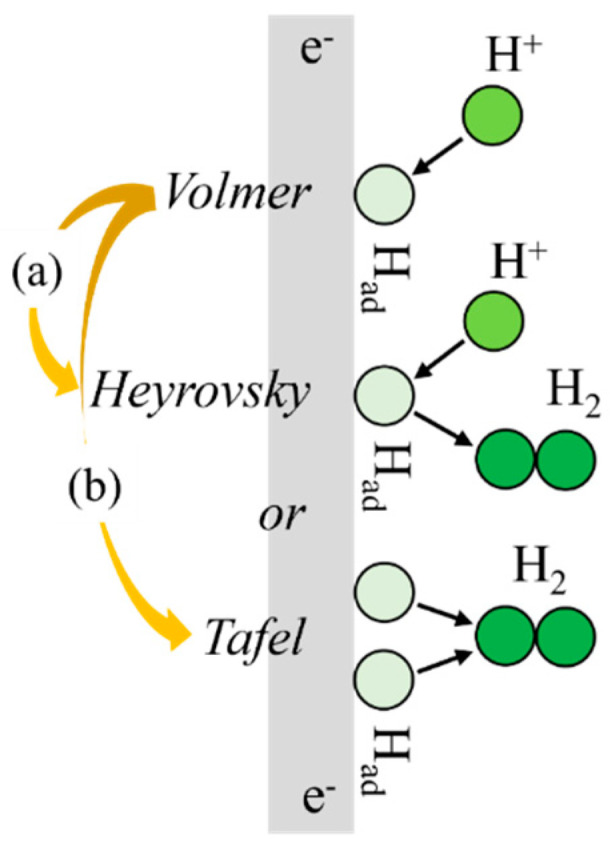
Schematic diagram of the steps of HER reaction: (a) Volmer–Heyrovsky mechanism and (b) Volmer–Tafel mechanisms.

2H_ad_ → H_2_ (Tafel reaction) (5)

where H_ad_ represents hydrogen adsorption. Typically, these reaction steps would occur on the electrode surface during hydrogen evolution. Hydrogen ions would be reduced and adsorbed on the electrode surface as hydrogen atoms (Volmer reaction). Subsequently, either one adsorbed atom would react with a solution hydrogen ion (Heyrovsky step) or a combination of two adsorbed atoms would lead to hydrogen evolution (Tafel reaction) (Figure 4) [32,33,34]. The process of the HER was influenced by the electrode potential and environmental factors. The HER could occur through two main mechanisms: the discharge–electrochemical mechanism (Volmer–Heyrovsky mechanism) (Equation (3) → Equation (4)) and the discharge–recombination mechanism (Volmer–Tafel mechanism) (Equation (3) → Equation (5)). During the HER, some of the hydrogen atoms adsorbed on the surface could be absorbed into the Ti alloy. The absorption of hydrogen atoms into the Ti alloy could result in HIC [35,36]. As evident from the reaction process, while some of the hydrogen atom generated at the cathode was released externally, a portion of it infiltrated the specimen, resulting in forced hydrogen injection.

### 2.3. Microstructural Assessment

Hydride formation in hydrogen-charged specimens was qualitatively analyzed using X-ray diffraction (XRD; SmartLab, Rigaku, Tokyo, Japan) after mechanical grinding and polishing. Then, the microstructure was observed using scanning electron microscopy (SEM; JSM-IT500, JEOL, Tokyo, Japan) after etching with Kroll’s solution (95 mL H_2_O + 3 mL HNO_3_ + 2 mL HF). Hydride formation in the hydrogen-charged specimens was observed using field emission SEM (FE-SEM; JSM-7100 F, JEOL, Tokyo, Japan) equipped with an electron backscatter diffractometer (EBSD; TSL Hikari Super, TSL, Mahwah, NJ, USA). The polished specimens underwent a final chemical–mechanical polishing process using colloidal silica for EBSD analysis. The orientation of each pixel was calculated from the measured Kikuchi diffraction patterns during EBSD analysis. To achieve a high confidence index (CI) for these orientations, the neighbor pattern averaging and reindexing (NPAR) technique was employed. This technique involved averaging the patterns of four neighboring points in a standard grid scan pattern. This process could effectively reduce the proportions of poorly indexed areas, thereby improving the accuracy of the analysis [37,38]. Finally, the analysis excluded orientations with a CI below 0.1.

### 2.4. Mechanical Tests

To analyze the microhardness along the surfaces and cross-sections of hydrogen-charged specimens, Vickers hardness tests using a microhardness tester (DK/Duramin-40 M1, Struers, Ballerup, Denmark) were performed. The microhardness of the surface of each specimen was measured by averaging ten different locations within a center point while applying a 300-gf load for 15 s. Conversely, the microhardness of the cross-section was tested under similar conditions at 50 μm intervals from the edge of the surface. To analyze the mechanical properties of hydrogen-charged specimens, compression tests were conducted using an Instron testing machine (Instron 4484, Instron, Norwood, MA, USA). The compression test specimens were machined to dimensions of 2.5 × 3.75 mm in a 1:1.5 ratio. The compression tests were performed at room temperature with a strain rate of 0.03/min.

## 3. Finite Element (FE) Modeling

### 3.1. Governing Equation

Fick’s law is employed to model the diffusion process, and time-dependent simulations can be obtained through Fick’s first law. In this study, Fick’s first law is specifically applied to describe steady-state diffusion, focusing on the spatial distribution of hydrogen under unidirectional diffusion. In an electrochemical cell system, hydrogen is adsorbed and absorbed at the cathode surface and then diffuses into the Ti alloy. The concentration of diffused hydrogen within the Ti alloy serves as a measure of the hydrogen flux. Assuming unidirectional diffusion, Fick’s law describes the diffusion with Equation (6) [39,40,41].

(6) $$J=-D\frac{\partial C(x,t)}{\partial x_{i}}$$

where *J* is the steady-state diffusion flux, C represents the hydrogen concentration, D is the diffusion coefficient, *x* is the distance from the surface along the thickness of the specimen, and $x_{i}$ represents the hydrogen charging direction.

The crucial factor in atomic movement is the hydrogen penetration depth from the experiment (d), defined as the effective displacement of hydrogen atoms during hydrogen penetration over time. During electrochemical hydrogen charging, some of the hydrogen atoms adsorbed on the metal surface are absorbed into the Ti alloys. The movement of these absorbed hydrogen atoms plays a significant role in determining the penetration depth. Therefore, the diffusion coefficient here can be referred to as the effective diffusion coefficient ($D_{eff}$). In one dimension, it is expressed as outlined in Equation (7) [42]. The discussion in Section 4.4 provides a detailed examination of diffusion.

(7) $$d=\sqrt{2D_{eff}t}$$

In this study, $D_{eff}$ is calculated using Equation (7) based on hydrogen penetration depth, obtained through microstructure and microhardness analyses.

### 3.2. Model Development

**Figure 5:**
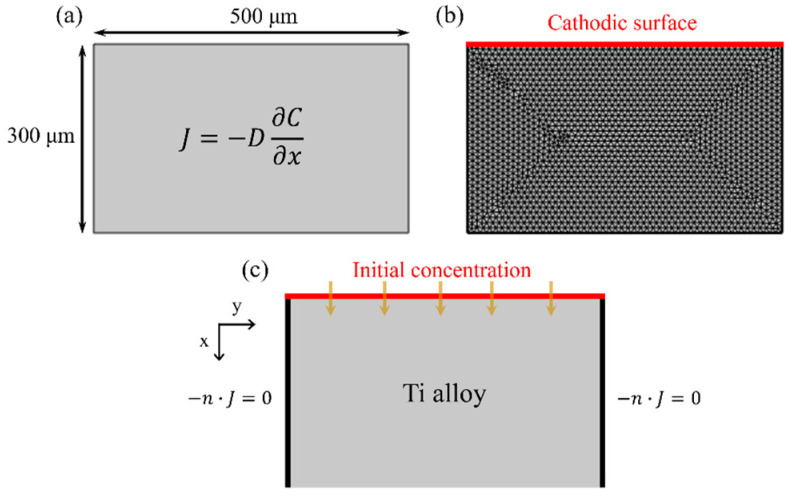
Schematic of the computational domain showing the (**a**) governing equation and dimensions, (**b**) mesh system, and (**c**) surfaces of each of the boundary conditions exposed to the electrolyte (0.5 M H_2_SO_4_) at room temperature. The red line represents the cathodic surface and initial concentration (1 mol/m^3^), which is the hydrogen charging surface. (The yellow arrows indicate the direction of hydrogen charging).

The 2D shape of the material domain is treated as near the surface of the specimen with a thickness of 300 µm. This results in domain dimensions of 300 µm × 500 µm, as depicted in Figure 5a. The resulting mesh comprises approximately ~4,000 adaptively refined triangular elements, as illustrated in Figure 5b. We employ an optimized geometry and mesh system.

### 3.3. Initial and Boundary Conditions

The necessary boundary conditions are displayed in Figure 5c. During the hydrogen charging simulation, it is assumed that the inflow boundary (cathodic surface) is exposed to a 0.5 M H_2_SO_4_ solution, which maintains a constant hydrogen concentration of 1 mol/m^3^. Left and right boundary surfaces are considered to have zero matter flux, with no flow assumed across the surfaces (Equation (8)). In Equation (8), n is the unit normal vector perpendicular to the boundary, used to define the direction of the flux at the boundary [43]. The direction perpendicular to the cathodic surface is assumed to have an infinite flux of matter, indicating an infinite diffusion of matter penetrated along the cathodic surface into the alloy. To support this assumption, the concentration at the bottom surface is set to zero, representing an infinite sink [44]. The results of the simulations of the hydrogen penetration under these boundary conditions are discussed in Section 4.4.

(8) −n·J=0

## 4. Results and Discussion

### 4.1. Microstructural Characteristics of the Uncharged Base Material

**Figure 6:**
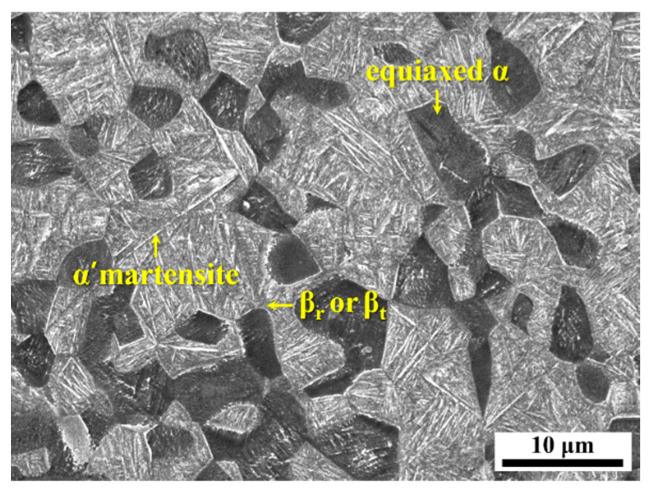
SEM micrographs of the surface of the uncharged specimen (0 h). (The yellow arrows indicate each phase).

SEM micrographs of the head region surface of the uncharged base material (referred to as 0 h throughout the study) are presented in Figure 6. Notably, the head region of the fastening bolt is the part typically exposed to the external environment in practical applications, making it the focus of this study. After solution treatment and subsequent rapid cooling (Figure 1), the prior β phase in the Ti64 alloy decomposes through an incomplete martensitic reaction. Therefore, the Ti64 alloy exhibits a final bimodal microstructure consisting of an equiaxed α phase and a transformed β phase (β_t_), as observed in the Figure. The β_t_ consists of α’ martensite and retained β phases (β_r_) [45,46,47,48].

**Figure 7:**
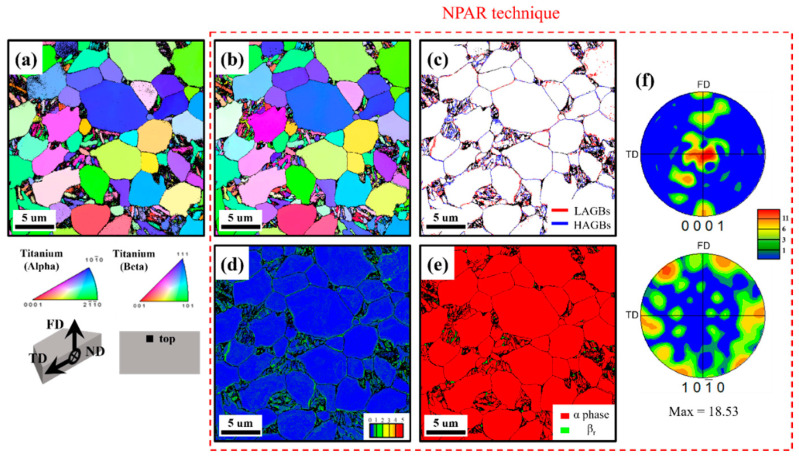
Microstructural features of top area in the head of the fastening bolt: before NPAR technique: (**a**) ND-IPF, after NPAR technique (red dotted rectangle): (**b**) ND-IPF, (**c**) grain boundary, (**d**) KAM, (**e**) phase map, and (**f**) basal plane {0001} and prismatic plane {10-10} pole figures.

**Figure 8:**
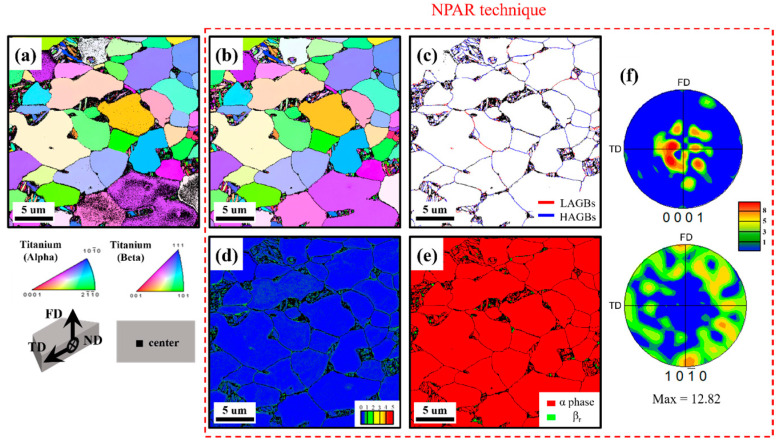
Microstructural features of center area in the head of the fastening bolt: before NPAR technique: (**a**) ND-IPF, after NPAR technique (red dotted rectangle): (**b**) ND-IPF, (**c**) grain boundary, (**d**) KAM, (**e**) phase map, and (**f**) basal plane {0001} and prismatic plane {10-10} pole figures.

For further confirmation, the microstructure of the top (near surface) and center (at 1.25 mm below the surface) areas in the head region of the 0 h specimen is initially analyzed using EBSD as presented in Figure 7 and Figure 8. The principal directions of the EBSD analysis are denoted as the normal direction (ND), transverse direction (TD), and forging direction (FD). Importantly, due to the intense deformation employed in the forging process, CI values below 0.1 are excluded from the EBSD analysis. This exclusion results in the dark regions observed in the EBSD maps in the figure. Therefore, the reliability of microstructural analysis is enhanced by using the NPAR technique to reduce zero solutions (dark areas with CI values below 0.1). The top area of the 0 h specimen shown in Figure 7a,b includes the inverse pole figure (IPF) maps before and after employing the NPAR technique. Comparing the results before (Figure 7a) and after (Figure 7b) applying the NPAR technique reveals that the zero solution decreased from 16.7 % to 8.7 %. The bimodal microstructure of the specimens is presented in Figure 7b, with an average grain size of 3.87 ± 1.93 μm. The EBSD results shown in Figure 7c–f are the results after applying the NPAR technique. The ratio of low-angle boundaries (LAGBs) to high-angle boundaries (HAGBs) in Figure 7c is 49.9 % and 50.1 %, respectively. The average kernel average misorientation (KAM) value is measured at 0.24 (Figure 7d), with higher KAM values concentrated along the grain boundaries. The phase map in Figure 7e predominantly consists of the α phase (99.4 %), indicating that the Ti alloy is primarily composed of the α phase. As previously mentioned, martensite forms during cooling, where the prior β phase transforms into β_t_, composed of martensite and β_r_ phases [49]. The martensite, possessing a similar lattice structure to the α phase, contributes to a total fraction of 99.4 %, as observed in Figure 7e [50]. Additionally, a basal texture (Figure 7f) developing in the head region of the fastening alloy can be attributed to the forging process mentioned earlier [51,52].

The center area of the 0 h specimens, as depicted in Figure 8a,b, also represents the results before and after employing the NPAR technique, where the zero solution decreased from 17.4% to 6.4%. A bimodal microstructure similar to Figure 8b is observed, with an average grain size of 4.70 ± 2.34 μm. Similarly, the EBSD results shown in Figure 8c–f are the results after applying the NPAR technique. In Figure 8c, the ratio of LAGBs to HAGBs is 33.1% and 66.9%, respectively. The average KAM value is measured at 0.16 (Figure 8d), with higher values concentrated along the grain boundaries. It can be observed that the top area presents a higher percentage of LAGBs (Figure 7c) and a higher average KAM value (Figure 7d) compared to the center, indicating more deformation occurred along the surface of the Ti alloy bolt head during the forging process [53]. In addition, similar to the top area, the phase map in Figure 8e is predominantly composed of the α phase (99.4%), with a texture formation observed due to the forging process. Here, the texture strength in the top area (Figure 7f) is influenced more by monotonic deformation, leading to higher texture in the top area compared to the center area [54]. The mechanical properties and diffusion simulations influenced by microstructural features and hydrogen uptake will be discussed in subsequent sections.

### 4.2. Microstructural Features of the Hydrogen-Charged Specimens

**Figure 9:**
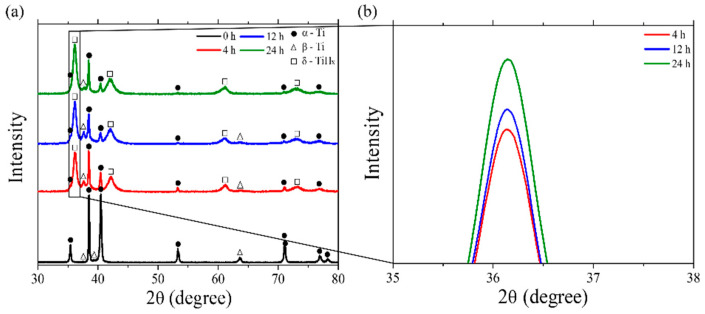
(**a**) XRD spectra of the charged Ti64 alloy with various charging times (0, 4, 12, and 24 h) and (**b**) comparison of δ-TiH_x_ peak intensity. Black rectangle shows the magnification of intensity in the range of 35–38 degrees.

The XRD results of the charged Ti alloy with various charging times (0, 4, 12, and 24 h) are presented in Figure 9, with a magnification of the intensity in the range of 35–38 degrees (black rectangle in Figure 9a shown in Figure 9b. The uncharged Ti alloy (0 h) exhibits high–intensity peaks corresponding to the Ti-α phase. Conversely, low peak intensities of Ti-β are observed, confirming the small amount of β_r_ phase in the microstructure. Through this, it can be further confirmed that the Ti alloy is predominantly composed of the α phase. After charging for 4 h, diffraction peaks of δ-TiH_x_ are observed. From Figure 9b, it is evident that the intensity of the δ-TiH_x_ peak increases with increasing charging time. This trend confirms the presence of hydrides in the hydrogen-charged specimens and shows that the intensities of the hydride peaks increase with the increasing hydrogen-charging time. These results indicate that hydrogen has successfully penetrated the Ti alloy, even under room temperature conditions. To confirm the presence and depth of the penetration of the hydride content in the specimens, microstructural analysis is conducted at a certain distance beneath the surfaces to determine the hydrogen penetration depth during charging.

**Figure 10:**
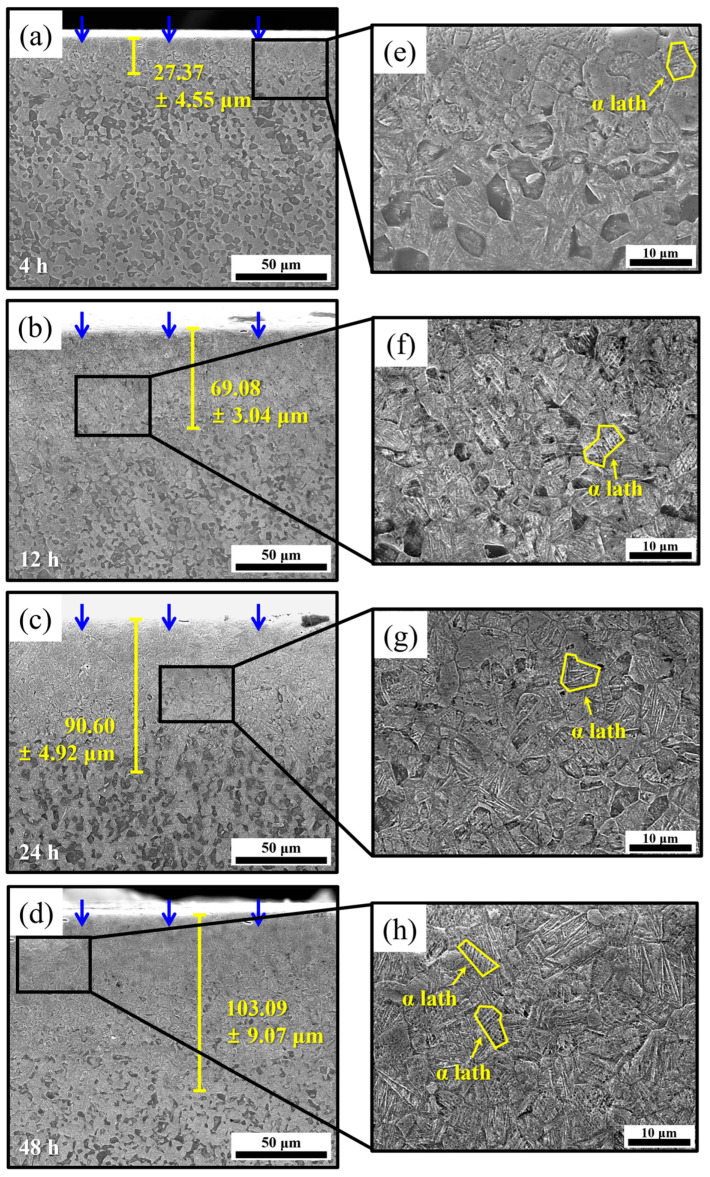
(**a**–**d**) Microstructural features of hydrogen-charged specimens after 4, 12, 24, and 48 h of hydrogen charging. (Blue arrows: hydrogen charging direction, yellow lines: hydrogen penetration depth where α laths are observed), and (**e**–**h**) magnified microstructures at regions’ hydrogen penetration depth.

**Figure 11:**
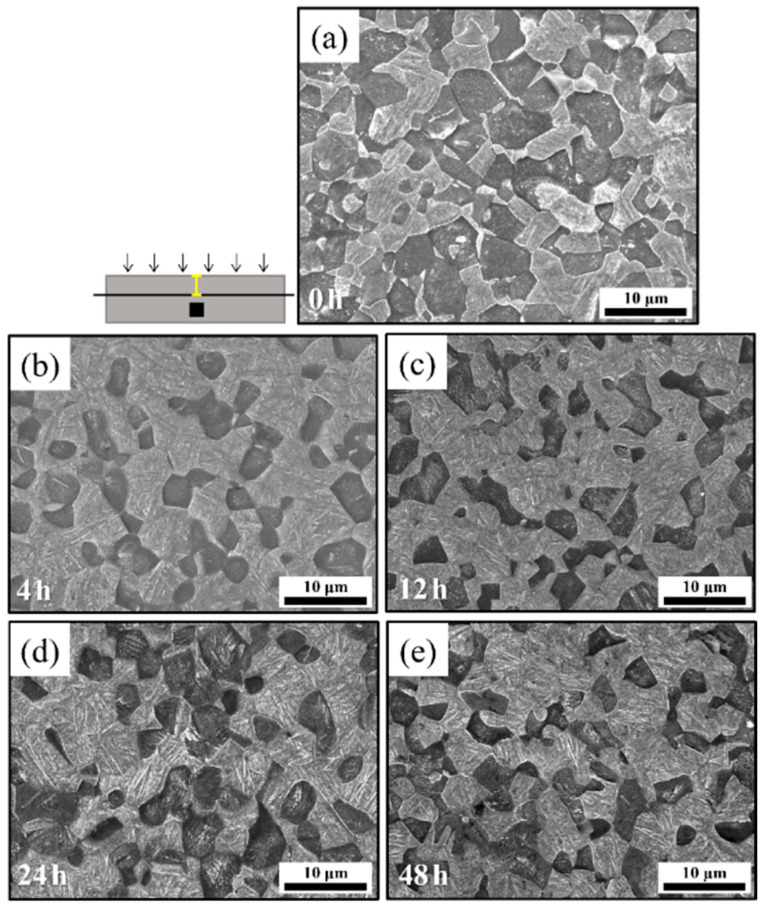
(**a**–**e**) Morphologies in the regions observed at distances beyond the hydrogen penetration depth (black rectangle) of charged specimens after hydrogen charging for 0, 4, 12, 24, and 48 h.

The microstructural results obtained from the cross-sectional analysis of the specimens charged with hydrogen for 0, 4, 12, 24, and 48 h are presented to assess the hydrogen penetration depth profiles for each charging time (Figure 10a–d). Magnified microstructures of the regions of the hydrogen penetration depth (yellow lines) for each charging time are shown in Figure 10e–h. The microstructures reveal the growth of α laths within the oversaturated Ti-α particle in regions below the surfaces of the hydrogen-charged specimens. This growth, resulting from hydrogen penetration and hydride formation even at room temperature, visibly demonstrates the regions influenced by hydrogen. To quantitatively confirm the areas where the microstructure changed due to hydrogen penetration, the depth was measured at 20 areas on each sample for each charging time where α laths appeared, and the average value was calculated and indicated with yellow lines (Figure 10a–d). It is observed that the growth areas of the α laths within Ti-α particles expand with increasing hydrogen charging times (Figure 10a–d). This observation is supported by the increased intensities of the hydride peaks in the XRD spectrum shown in Figure 9b. In addition, the microstructural analysis of regions at distances beyond the hydrogen penetration depth (Figure 10a–d) is presented in Figure 11a–e. Figure 11a corresponds to the specimen without hydrogen charging; as described previously (Figure 7 and Figure 8), it exhibits distinct equiaxed α and β_t_ phases. Additionally, the microstructures observed in these regions of the hydrogen-charged specimens (Figure 11b–e) are similar to those of the uncharged specimen. Consequently, the microstructural changes are less prominent at distances farther from the surfaces of the charged specimens, potentially due to the low hydrogen diffusion level at room temperature.

**Figure 12:**
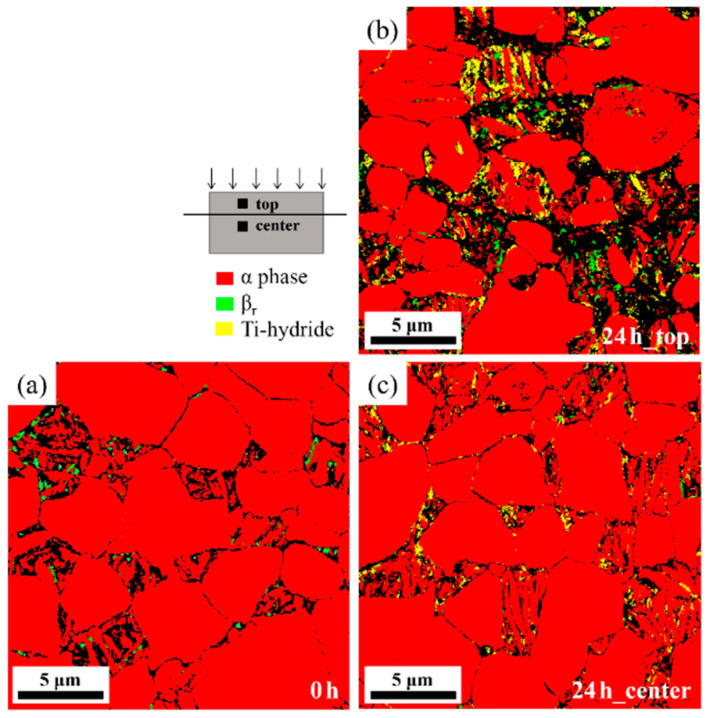
Microstructural features in phase map of Ti64 alloy for electrochemical hydrogen charging after NPAR: (**a**) uncharged specimen and (**b**) top and (**c**) center areas of the specimen with hydrogen charging. (The red, green, and yellow colors represent the Ti-α phase, β_r_ phase, and Ti-hydride, respectively).

In addition, a quantitative analysis of hydrides in the hydrogen-charged specimens is conducted using EBSD. The uncharged specimen reveals that the Ti-α phase constitutes 99.3% and the β_r_ phase constitutes 0.7% of the Ti alloy. The microstructure of the region below the surface of the specimen charged for 24 h (Figure 12b: top) shows that the Ti-α phase accounts for 93.0% and the β_r_ phase accounts for 1.2%, with an additional Ti-hydride phase being observed at 5.8%. Furthermore, the microstructure of the region farther from the surface of the specimen charged for 24 h (Figure 12c: center) shows that the Ti-α phase constitutes 97.8%, the β_r_ phase constitutes 0.2%, and the Ti-hydride phase accounts for 2.0%. The presence of hydrogenation in the charged specimens is attributed to the diffusion of hydrogen, forming hydrides during prolonged hydrogen charging. This EBSD analysis confirms the predominant presence of hydrides in both grain boundaries and within the grains while enabling quantitative comparisons.

### 4.3. Mechanical Properties

#### 4.3.1. Microhardness Assessment of Uncharged and Charged Specimens

**Figure 13:**
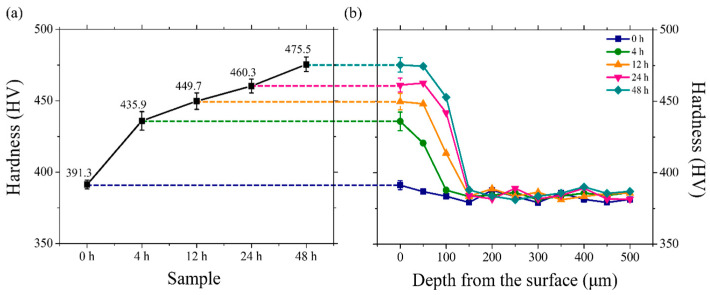
(**a**) Variations in Vickers hardness values of surface of Ti64 alloy with hydrogen charging time (0, 4, 12, 24, and 48 h), and (**b**) Vickers hardness values as a function of depth from the surface with respect to the charging time.

To analyze and confirm the impact of hydrogen, the hardness levels of the regions exhibiting microstructural changes after hydrogen charging are examined using a Vickers hardness tester. The hardness profile displaying the average values and deviations of the surface microhardness values of hydrogen-charged specimens is shown in Figure 13 and summarized in Table 2. As the hydrogen-charging time increases, an increase in hardness is observed. This increase can be attributed to solid–solution strengthening and precipitation-strengthening effects [55,56,57,58,59]. For solid–solution strengthening, more hydrogen atoms are dissolved in the matrix as the charging time increases, leading to enhanced reinforcement effects. Precipitation-strengthening results from the diffusion and enrichment of the hydrogen atoms in the specimen as charging time increases. This phenomenon forms brittle hydrides, subsequently enhancing the hardness of the specimen [19,22]. This phenomenon can be attributed to the influences of the penetration depths due to hydrogen charging. The region where microstructural changes are observed (indicated by the yellow lines in Figure 10a–d) shows evidence of precipitation strengthening. Beyond this depth, in areas where no microstructural changes are observed, the effects of hydrogen penetration appear to result from solid–solution strengthening.

#### 4.3.2. Uniaxial Compression Behaviors of the Uncharged and Charged Specimens

**Figure 14:**
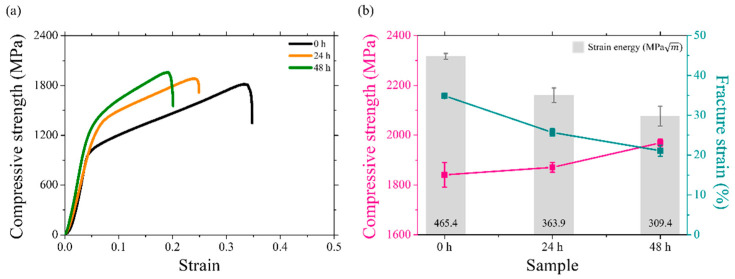
(**a**) Engineering stress–strain curves of the hydrogen-charged specimens and (**b**) plot of the mean values and standard deviations for the mechanical properties including compressive strength, fracture strain, and strain energy.

The results of the compression tests conducted to observe the influences of hydrogen charging on the mechanical behaviors of Ti alloys are presented (Figure 14a). The stress–strain curves of the uncharged and hydrogen-charged specimens after 24 and 48 h are shown and compared. Compression tests are conducted three times to ensure reproducibility. The values of the mechanical properties, including compressive strength, fracture strain, and strain energy, are averaged and presented with their respective deviations in Figure 14b. The compressive strength increases with increasing hydrogen charging time. The specimens charged with hydrogen exhibit significantly higher compressive strengths than the uncharged specimen. This trend can be attributed to the solid–solution strengthening and precipitation-strengthening effects of hydrogen within the Ti matrix [44]. Microstructural evidence and the controlled experimental conditions in this study suggest specific mechanisms that may contribute to this unique behavior. The influences of hydrogen on Ti alloys reportedly increase the rate of nucleation of dislocations within the microstructure of the alloy [19]. Additionally, diffusible hydrogen accumulated around dislocations hinders dislocation slip by following their motion, thereby increasing the compressive strength with increasing hydrogen concentration [4,19,21,60]. Conversely, a decrease in fracture strain is observed with increasing hydrogen charging time. When evaluating strain energy, the specimens decrease in strain energy as the hydrogen charging time increases, indicating the characteristic feature of HIC. This finding can be attributed to the precipitation of hydrides during compression deformation, causing premature failure associated with a ductile-to-brittle transition [45].

### 4.4. Predicted Hydrogen Diffusion Behavior by the FE Model

In this part of the study, the hydrogen penetration depth obtained from the analyses of microstructure and microhardness is employed to calculate $D_{eff}$. To effectively incorporate the experimental effects of hydrogen penetration into the modeling, $D_{eff}$ is specifically calculated. Through this, numerical simulations are implemented to predict the hydrogen penetration depth when exposed to a hydrogen environment for longer periods.

The discussion of the diffusion in polycrystalline materials is centered primarily around two interrelated mechanisms: diffusion through the lattice and diffusion along the grain boundaries. In the case of most metals, diffusion occurs through atomic migration driven by defects such as dislocations that may exist at the grain boundaries or through atomic migration in vacancies and interstitial spaces [61,62,63]. For instance, the grain boundary and lattice diffusion coefficients of α-Ti alloy are reported to be 9.1 × 10^−9^ m^2^/s and 2.6 × 10^−14^ m^2^/s, respectively [64]. Therefore, to determine the $D_{eff}$ for each charging time (Table 3), Equation (7) is employed using data obtained from the microstructural and microhardness analyses. The results confirm that lattice diffusion is the dominant diffusion path for the $D_{eff}$ for each charging time listed in Table 3. Comparison of the $D_{eff}$ for each charging time shows that a relatively low $D_{eff}$ is observed at the initial time of 4 h and at a later time of 48 h. In established studies, the presence of a thin TiO_2_ passive film on uncharged Ti alloys has been frequently noted [65,66]. Due to how thin the passive film is, it was not observed during the XRD analysis (Figure 9a). However, the reported hydrogen diffusion coefficient in TiO_2_ film is significantly lower than the lattice diffusion of Ti-α phase [67]. Thus, it can be correlated with the low $D_{eff}$ at the relatively early time of 4 h, when the hydrogen diffusion into TiO_2_ layer is likely to be impeded. This behavior reflects the inherent resistance of the TiO_2_ passive film to hydrogen diffusion, aligning with its reported diffusion properties, and underscores its influence during the initial stages of hydrogen charging. At 12 and 24 h, a similar $D_{eff}$ is observed for both times, indicating that hydrogen diffusion proceeds consistently during prolonged hydrogen charging. However, there is a tendency for $D_{eff}$ at 48 h to decrease.

As discussed earlier, the head region of the fastening alloy undergoes severe plastic deformation processes; thus, lattice defects such as grain boundaries and dislocations are introduced into the microstructure. These features act as pathways for hydrogen diffusion, hence enhancing the hydrogen diffusion within the microstructure [68,69,70]. As seen in Figure 7 and Figure 8, due to the forging process, the fraction of LAGBs decreases as the distance from the surface increases. This trend continues until reaching the central region, as illustrated in the figures. Consequently, it can be inferred that $D_{eff}$ at 48 h decreases due to the decreased grain boundaries and dislocation densities in that region. This decrease suggests that while lattice diffusion dominates overall, the reduced contribution from grain boundary pathways at 48 h plays a notable role in the observed trend. This indicates that although lattice diffusion is the overall dominant diffusion path, grain boundary diffusion has to be included to some extent.

**Figure 15:**
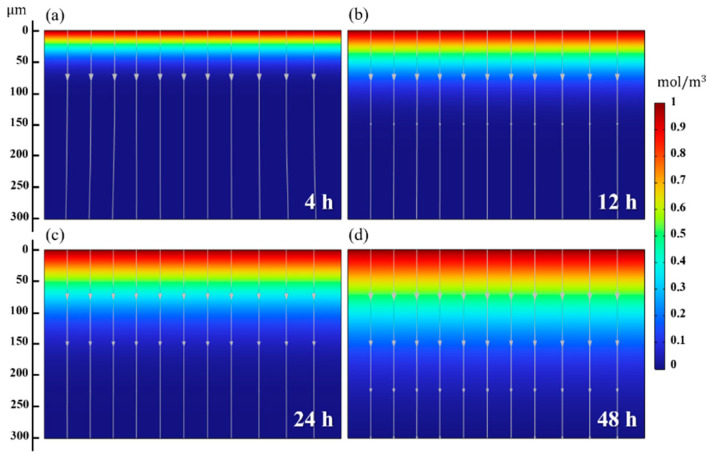
(**a**–**d**) Distribution of hydrogen concentration (color) in the Ti alloy at the different charging times (4 h, 12 h, 24 h, and 48 h). The color in the legend corresponds to the changes in the molar concentration of hydrogen in mol/m^3^ with an initial concentration of 1 mol/m^3^ at cathodic surface. Streamlines show diffusion direction within the alloy.

**Figure 16:**
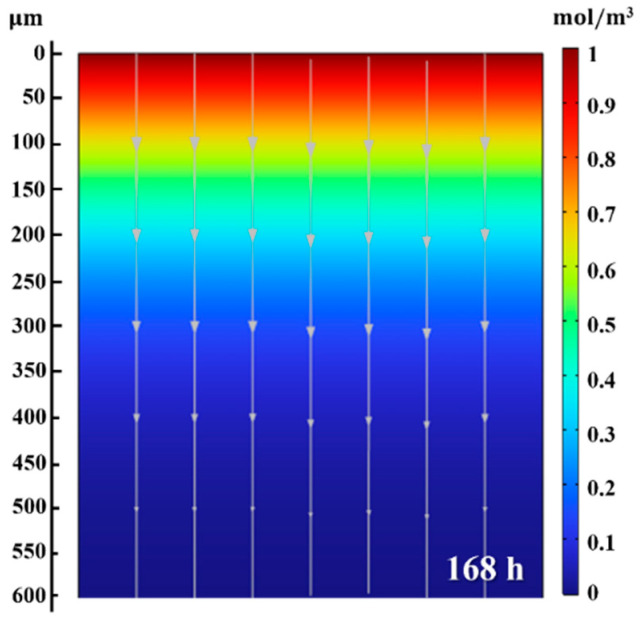
Distribution of hydrogen concentration (color) in the Ti alloy at 168 h. The color in the legend corresponds to the changes in the molar concentration of hydrogen in mol/m^3^ with an initial concentration of 1 mol/m^3^ at cathodic surface. Streamlines show diffusion direction within the alloy.

Using the lattice diffusion coefficients, $D_{eff},$ for the four charging conditions, observations and predictions of the hydrogen penetration depth can be made. The analysis of diffusion behavior in simulation employs the average value of $D_{eff}$ (4.1 × 10^−14^ m^2^/s) for each charging time. Essentially, assuming constant mobility, the extent of the hydrogen diffusion is confirmed by assessing the hydrogen penetration depth within the Ti alloy. The numerical simulation results are depicted in Figure 15a–d, and upon comparison with Figure 10, it is observed that microstructural changes occur in regions where the concentration ≥ 0.4 mol/m^3^ for all charging times. In addition, the regions with <0.4 mol/m^3^ concentration are identified to contain hydrogen atoms which contribute to solid–solution strengthening, without any microstructural changes. Simulations for longer periods, such as 168 h (7 days), are conducted (Figure 16) to predict the effect of the hydrogen charging after lengthy periods. The hydrogen penetration depth is expected to be approximately 500 μm, as indicated by the arrow at the end of the streamline, signifying the end of hydrogen penetration depth. Also, microstructural changes due to hydride formation are anticipated, up to a maximum depth of about 200 μm (≥ 0.4 mol/m^3^). This analysis showcases a significant comprehension of the relationship between the results of hydrogen penetration depth and areas sensitive to HIC occurrence.

## 5. Conclusions

In this study, cathodic electrochemical hydrogen charging was used to investigate the microstructures and mechanical properties of Ti64 alloy fastening components. The presence of hydrides, confirmed through XRD, SEM, and EBSD analyses, demonstrated that hydride formation and hydrogen penetration depth increased with charging time. Notably, microstructural changes were observed even at room temperature and under relatively short charging durations, enabling a clear analysis of hydrogen penetration depth.

Vickers hardness tests revealed increased surface and cross-sectional hardness due to solid–solution and precipitation strengthening, correlating with the hydrogen-charged layers observed in the microstructure. Compression tests showed an increase in compressive strength but a decrease in fracture strain and strain energy, highlighting the detrimental effects of hydrogen charging on mechanical reliability due to the accumulation of brittle hydrides.

Based on microstructural and hardness data, the average diffusion coefficient was calculated, and numerical simulations were used to predict hydrogen penetration depth and regions prone to embrittlement. The strong correlation between experimental results and simulations underscores the importance of understanding hydrogen diffusion mechanisms to predict penetration behavior and mitigate hydrogen-induced cracking. These findings provide valuable insights for designing hydrogen-resistant materials and ensuring their reliability in practical applications.

## Figures and Tables

**Table 1:** Chemical compositions of Ti64 alloy used in the present study (in wt.%).

	N	C	H	Fe	O	Al	V	Y	Ti
Ti64	0.002	0.010	0.004	0.210	0.190	6.4	3.95	0.001	Bal.

**Table 2:** Microhardness values of Ti64 alloy with various charging times from 0 h to 48 h.

Specimen	Microhardness (HV)
0 h	391.3 ± 3.1
4 h	435.9 ± 6.5
12 h	449.7 ± 5.8
24 h	460.3 ± 4.8
48 h	475.5 ± 5.1

**Table 3:** Microhardness values of Ti64 alloy with various charging times from 0 h to 48 h.

Specimen	Hydrogen Penetration Depth (Figure 10a–d) (μm)	$D_{eff}$ (m^2^/s)
4 h	27.37 ± 4.55	2.61 × 10^−14^
12 h	69.08 ± 3.04	5.53 × 10^−14^
24 h	90.60 ± 4.92	5.33 × 10^−14^
48 h	103.09 ± 9.07	3.08 × 10^−14^

## Data Availability

The raw/processed data required to reproduce these findings cannot be shared at this time as the data also form part of an ongoing study.
